# Job Performance of Medical Graduates With Compulsory Services in Underserved Rural Areas in China: A Cohort Study

**DOI:** 10.34172/ijhpm.2022.6335

**Published:** 2022-02-14

**Authors:** Mingyue Li, Ziyue Wang, Baisong Zhang, Tiantian Wei, Dan Hu, Xiaoyun Liu

**Affiliations:** ^1^Department of Health Policy and Management, School of Public Health, Peking University, Beijing, China.; ^2^China Center for Health Development Studies, Peking University, Beijing, China.

**Keywords:** Health Workforce, Medical Education, Compulsory Services Programs, Job Performance

## Abstract

**Background:** China started a national program in 2010 to train qualified general practitioners with compulsory services program (CSP) in rural and remote areas. While the program has shown positive effects on staffing primary healthcare (PHC) in rural areas, very little is known about how well they perform. This study aims to evaluate the job performance of medical graduates from this program and the influence of program design on job performance.

**Methods:** A cohort study was conducted with graduates from CSP and non-CSP (NCSP) from four medical universities in central and western China. Baseline and three waves of follow-up surveys were conducted from 2015-2020. The pass rate of China National Medical Licensing Examinations (NMLE) and self-reported job performance were used as measurements. Multivariable regressions were used to identify factors affecting job performance.

**Results:** 2154 medical graduates were included, with 1586 CSP and 568 NCSP graduates. CSP (90.6%) and NCSP (87.5%) graduates showed no difference in passing the NMLE (*P*=.153). CSP graduates reported similar job performance with NCSP graduates (CSP, 63.7; NCSP, 64.2); in the multivariable regression, CSP graduates scored 0.32 and 1.36 points lower in the total sample and graduates of 2015-2017, respectively, but not significantly. Having formally funded positions improved the job performance of CSP (β coefficient=4.87, *P*<.05). After controlling for Qinghai which adopted a different contracting strategy, "working in hometown" showed significant influence on job performance (β coefficient = 1.48, *P*<.05).

**Conclusion:** CSP graduates have demonstrated as good job performance as NCSP, proving the competency to provide high-quality care for remote and rural areas. The contracted township health centers (THCs) should provide guidance for CSP graduates, especially in the first few years after graduation. The local government should provide formally funded positions on time and prioritize signing contracts with hometowns or places nearby.

## Background

**Key Messages**

** Implications for policy makers**

- The implementation of compulsory services program (CSP) achieved success in providing more medical graduates to rural and remote areas in China, but allowing CSP graduates to choose signing places by rank of score could undermine their job performance.
- The design of the policy, including formally funded positions and signing contracts with hometowns, could have beneficial influence on medical graduates in the program.
- Early career and life guidance should be provided who to young CSP graduates working in rural settings, especially those who presented lower job performance compared.

** Implications for the public**

The inequitable health workforce distribution in China demands more attention. Compulsory services programs (CSPs) have been implemented globally as a strategy to redistribute health workforce. After training medical graduates for rural areas, the government needs to make sure they have the capability to provide high-quality healthcare services to rural population. Formally funded positions, working in hometowns, and early career and life guidance should be provided with good supervision. The government should invest in the health workforce for rural and remote areas so that CSP graduates can have high job performance and still pursue careers even not in city-based hospitals.

One of the most challenging tasks of policy-makers is to provide people living in rural and remote areas access to affordable care from well-trained health workers.^1-3^ This problem is common to almost all countries and poses a major challenge to the nationwide provision of equitable health services.^4-6^ In 2010, the World Health Organization (WHO) issued global policy recommendations focusing on increasing access to health workers in remote and rural areas.^1^ Countries around the world have explored and implemented a variety of interventions to train, attract, and retain health workers for rural areas,^7-13^ including compulsory services programs (CSPs).^14-16^ Since the early 20th century, CSP have been implemented in many countries,^14^ and are deemed a way of pursing social justice and health equity, because they enable governments to allocate health resources to areas that are scarce in resources and to communities that are discarded by market forces. However, very little is known about the effectiveness of CSP.^17^ High job performance is crucial for health workers to provide quality care, thereby strengthening health systems and improving health outcomes.^18,19^ If CSP could improve health worker supply for rural areas but with inferior job performance and service quality, it would lead to the problems of health inequity. However, most studies focused on attraction and retention of health workers for rural areas,^20-22^ few, if any, paid attention to how those retained health workers perform in rural areas.^23^ One previous study found that the clinical competencies of special track graduates (similar to CSP) scored higher than those of normal track (similar to non-CSP – NCSP) in Thailand using self-assessment questionnaires.^24^

China has a three tiered healthcare delivery system in rural area, county hospitals, township health centers (THCs), and village clinics (VCs).^25^ China’s THCs and VCs are the main providers of primary healthcare (PHC). People can go to any health institutions freely, but they will get more reimbursement if they were referred to upper-level hospitals from primary health institutions.^26^ China’s PHC, providing basic care and public health services to 1.4 billion population, faces great challenges in its capacity and equity.^27^ One of the biggest challenges is the inadequate qualified health workers and insufficient competence of current workforce in primary care. In 2020, 32% of the health workers work in PHC; 29% of licensed and assistant licensed doctors work in PHC.^28^ In 2019, 82.6% health professionals at THC received less than a junior medical college level of education. Physicians at hospitals have more opportunities to see different patients and receive in-service trainings than those at PHC, which contributes to low performance of PHC physicians. Residents have little trust on the quality of PHC services.^28^ As a result, most residents bypass PHC in favor of costly higher hospitals for even minor conditions.^29^ From 2010 to 2019, the percentage of outpatient and inpatients visits of PHC facilities to all healthcare facilities has decreased year by year.^30^ In rural and remote areas, this problem is worse because more health workers are concentrated in urban and wealthier areas.

A series of health system reforms were launched to strengthen the capability of primary care.^31^ In order to enhance the capability of rural PHC and increase health accessibility and equity, in 2010, China started a national CSP for medical students, training general practitioners for rural areas in central and western regions.^32^ Medical universities mainly recruit students with rural backgrounds. The admission score of CSP students in the university entrance examination is usually 20 points lower than that of NCSP peers, raising a concern on the inferior quality of CSP students and their job performance.^33^ On matriculation, students need to sign contracts with local health administrations and medical universities, and commit to go to the appointed THCs or VCs to practice for six years soon after five-year undergraduate medical education. Students do not need to pay for tuition or accommodation, and can receive monthly allowances. The medical education curriculum is similar for the two programs, except that the CSP students have rural and community health courses and internship in PHC settings. Like NCSP students, CSP students need to complete a rotating internship in tertiary hospital, but they also need to go to secondary hospital and primary health facility to complete internship in the five-year undergraduate study.^33^ The teaching hospital provide specialty rotation, including training in case consultation, writing medical records, basic clinical skills and related health policies. The THCs and community health centers provide training in common disease diagnosis and treatment in PHC and medical regulations. CSP students need to take the evaluations after each internship period and also a final examination before graduation. The county which the CSP graduates are required to sign contract with are decided by local government depending on the local demand and supply of health workforce. Preliminary evaluations have shown that the majority of medical graduates in this program can fulfill the contract to work in a rural area.^34-36^ It has been five years since the first wave of students graduating from CSPs. More than 5000 medical students graduate from this program each year in China. Much still needs to be understood about their competency and how they perform in rural and remote areas and how the design of CSP have influenced their performance.

 In this paper, we examined the job performance of medical students graduating from CSP in four medical universities in China, and compared with their school peers (NCSP). This study can take us a step further in understanding the implementation and the effect of recruiting and retaining health workers for underserved areas through CSP, and provides evidence for other countries that are also seeking to increase access to health workers in underserved areas.

## Methods

###  Study Design and Data Collection 

 Data used in this paper were retrieved from Cohort Study of Medical Graduates with Compulsory Services in Rural Areas Studies, funded by China Medical Board. The study was launched in 2015 and has established five cohorts in five years of medical graduates. All participants provided informed consent. A nonrandom purposive sample of four medical universities which undertook CSP were chosen from western and central China, representing middle- and low-level economic regions. The survey included 3620 medical graduates from Qinghai University (located in Qinghai province, northwest China), Guangxi Medical Universities (located in Guangxi Zhuang autonomous region, southwest China), Jiujiang University and Gannan Medical University (located in Jiangxi province, central China). The location of three provinces and four universities in China is presented in Figure, with the initial cohort size.

**Figure:**
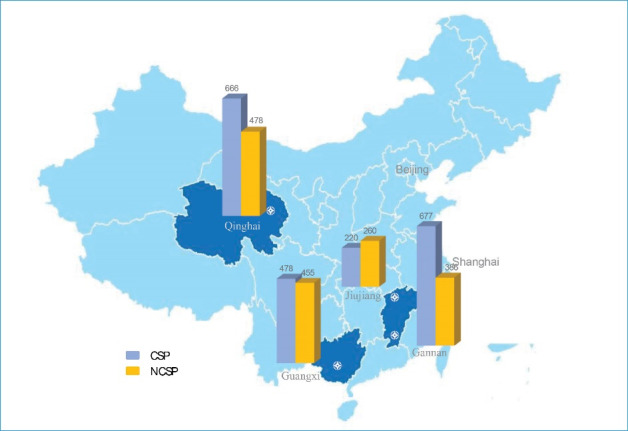
Locations of Four Medical Universities in China and Initial Cohort Size. Abbreviations: CSP, compulsory services program; NCSP, non-compulsory services program.

 There are two types of medical graduates in this study, those who are required to practice in rural and remote areas after graduation (CSP) as the intervention group, and common medical graduates (NCSP) as the comparison group. In each university, all CSP classes were included in the study and were matched with corresponding same-year NCSP classes at 1:1 ratio.

Self-administered questionnaire surveys were conducted on site to collect baseline data before the students graduate, with informed consent. Online followed up surveys were then conducted every year after graduation. We used a well-established online survey platform (https://www.wjx.cn) to organize the follow-up surveys. Instant messaging app (WeChat), telephone call, and liaison key persons were used to facilitate the survey and improve the response rate. Four round of follow-up surveys had been conducted in 2016, 2017, 2018, and 2020. Information of demographic characteristics, employment, postgraduate study, residency training and job-related information for the two types of graduates were collected.

###  Measurement: Pass Rate of National Medical Licensing Examinations 

In China, the National Medical Licensing Examinations (NMLE) has been implemented for 20 years since the 1999 Law on Practicing Doctors.^37^ The NMLE is sponsored by China National Medical Examination Center, an affiliated institution of National Health Commission. All practicing doctors in China must pass the NMLE before they can register as a licensed doctor or a licensed assistant doctor in order to practice legally. The NMLE includes clinical skill test and general medical knowledge test, which can well reflect candidates’ professional competency.^38^ Medical licensing examination has been linked with job performance in previous studies.^39-41^ Medical licensing examination scores were proved to be significant predictors of clinical competence and quality of care.^40^ We adopted the pass rate of NMLE as an objective indicator of CSP graduates’ job performance, and compared with that of NCSP graduates.

###  Measurement: Job Performance Scale 

Conceptual grounds for job performance can be found in well-established theories of human capital and psychology.^42^ Job performance was defined as the “aggregated value to the organization of discrete behavior episodes that an individual performs over a standard interval of time.”^43^ To our knowledge, job performance is generally measured from two dimensions, including task performance and contextual performance. Measuring job performance by scale has been proved reliable and consistent compared with results from that measured by objective indicators.^44^ Most scales were designed from task and contextual dimensions before. However, learning capability for employees has been attached to more importance in job performance studies, and recent scales have included this dimension.^45,46^ The scale we adopted included measurements of all three dimensions of job performance.^47^ This scale was based upon widely-used international job performance scale and revised in adaptation to Chinese context. It has been used in studies on rural health workers and GPs’ job performance and has proved valid cultural adaptation.^48-50^

The self-rated job performance scale included three dimensions and twelve items (task performance: four items; contextual performance: five items; learning performance: three items). The definition for each dimension is provided (see Table S1, Supplementary file 1). The items are rated on a 7-point Likert scale of how often the behavior or feeling, or attitude has been manifested. The total score ranges from 1 (lowest job performance) to 84 (highest job performance). One’s total job performance score was obtained by adding the scores of each item. Score for each dimension was also calculated. The alpha internal consistency coefficient of reliability was 0.89 in this study, indicating that the items were highly internally consistent. The details of the dimensions, definitions, and reliability of the job performance scale was provided in supplementary file (see Table S1, Supplementary file 1). The validity was previously examined using confirmatory factor analysis with absolute fit indices, incremental fit indices and parsimony fit indices, and all the goodness-of-fit indices showed good content validity.^47^

###  Statistical Analysis 

Descriptive analysis was used to identify characteristics of the sample. Percentages were calculated for qualitative data, and means and standard deviations (SDs) for quantitative data. For pass rate of NMLE, chi-square test was used to compare the differences between CSP and NCSP graduates. For job performance scale, we determined the total scores of job performance scale for CSP and NCSP graduates separately by different demographic and job-related variables. One-way analysis of variance (ANOVA) was used to examine the differences among these characteristics. One-sided *t *test was used to compare the scores of job performance scale between the two groups (CSP vs. NCSP). The *P *value below.05 was considered statistically significant.

The job-related factors, including obtaining formally funded positions (which means these are tenure positions with full salary support, secured pensions and other benefits from government budget, and are considered as formal employees of Chinese public sectors) becoming attending physicians, working in hometown district, all showed significant difference for CSP graduates in ANOVA, so we further fitted multivariable linear regression models to explore the association between the design of the program and job performance for CSP graduates. Total job performance scores were used as the dependent variable. Since some graduates of 2018 and 2019 are still based in tertiary hospitals for residency training in the 2020 follow-up survey, and graduates of 2015-2017 have finished the 3-year residency and returned to contracted organizations, we restricted the sample in graduates of 2015-2017. We also conducted robustness check to verify our findings. Firstly, we used logarithm of total performance score for regression, with coefficients and standard errors reported. Secondly, a dichotomous indicator of job performance variable was used for logistic regression (those obtaining 6 or 7 for each item with the total score being or above 72 were identified as high job performance and was coded as “1,” otherwise coded as “0”). Odds ratios and corresponding 95% confidence intervals were reported (see Supplementary file 2). Lastly, the three sub-dimensions of job performance, score of task, contextual and learning performance were replaced as the dependent variables for regression (see Supplementary file 2). All statistical analyses were conducted in Stata 15.1 (StataCorp LP, College Station).

## Results

###  Basic Characteristics of the Cohort 

Around 300-400 students were included in each baseline survey from 2015 to 2019 in the five cohorts. The response rate was higher for CSP than NCSP across cohorts (see Table S2, Supplementary file 1). Table 1 shows the summary statistics of the sample by CSP and NCSP graduates, including medical universities, years of graduation, demographic characteristics, current workplace, and job-related information. The sample used for analysis in this study were limited to those who are currently working, otherwise they cannot answer the job performance scale in the questionnaires, for example, those who are undertaking postgraduate study. The total sample amounts to 2154, including 1586 CSP, accounting for 73.63% (Table 1). The majority of CSP (n = 11546, 97.48%) have obtained formally funded positions, while only 33.63% (n = 1191) of NCSP have got the formally funded positions. The average monthly income of graduates was 3883 CNY (SD = 13379) per month in CSP graduates, and 5903 CNY (SD = 15397) per month in NCSP graduates. Most graduates of CSP work in the contract signing places (n = 11551, 97.8%), but only 59.6% (n = 1945) of them work in their hometown. Although they have graduated 5 years at most, some graduates of CSP (n = 1113, 7.1%) have been promoted to attending physicians.

**Table 1:** Summary Statistics of the Sample, No. (%)

**Variables**	**CSP (n = 1586)**	**NCSP (n = 568)**	**Overall (n = 2154)**
Schools			
Qinghai	504 (31.78%)	166 (29.23%)	670 (31.10%)
Guangxi	408 (25.73%)	173 (30.46%)	581 (26.97%)
Jiujiang	181 (11.41%)	117 (20.60%)	298 (13.83%)
Gannan	493 (31.08%)	112 (19.72%)	605 (28.09%)
Years of Graduation			
2015	219 (13.81%)	152 (26.76%)	371 (17.22%)
2016	326 (20.55%)	126 (22.18%)	452 (20.98%)
2017	377 (23.77%)	122 (21.48%)	499 (23.17%)
2018	317 (19.99%)	90 (15.85%)	407 (18.90%)
2019	347 (21.88%)	78 (13.73%)	425 (19.73%)
Demographic Characteristics			
Female	762 (48.05%)	294 (51.76%)	1056 (49.03%)
Male	824 (51.95%)	274 (48.24%)	1098 (50.97%)
Married	498 (31.40%)	178 (31.34%)	676 (31.38%)
Not married	1088 (68.60%)	390 (68.66%)	1478 (68.62%)
Current Workplace			
Public hospitals at county level and above	131 (8.26%)	510 (89.79%)	641 (29.76%)
CHC & THC	1446 (91.17%)	12 (2.11%)	1458 (67.69%)
Other	9 (0.57%)	46 (8.10%)	55 (2.55%)
Current Job Information			
Obtaining formally funded positions	1546 (97.48%)	191 (33.63%)	1737 (80.64%)
Income of current job/month, Mean (SD)	3883 (3379)	5903 (5397)	4416(4107)
Working in contract signing place	1551 (97.79%)	-	1551 (72.01%)
Working in hometown district	945 (59.58%)	-	945 (43.87%)
Becoming attending physicians	113 (7.12%)	2 (0.35%)	115 (5.34%)

Abbreviations: CSP, compulsory services program; NCSP, non-compulsory services program; CHC, community health center; THC, township health center; SD, standard deviation.

###  Results of Pass Rate of China National Medical Licensing Examinations 

Table 2 displays the results of the pass rate of NMLE by CSP and NCSP and by medical universities. The pass rate was 90.6% in CSP graduates, and 87.5% in NCSP graduates. Overall, CSP graduates showed no difference in the NMLE compared with NCSP graduates (*P*= 1.153). In Qinghai, Guangxi, and Jiujiang, CSP graduates all reported higher rates of passing the examination than their counterparts, and did not show statistically significant difference with NCSP graduates. 76.8% (n = 1268) of Qinghai CSP graduates passed the NMLE, and 76.5% (n = 1101) of NCSP graduates passed this qualification exam (*P*= 1.949). In Guangxi, 98.4% (n = 1312) of CSP graduates and 96.7% (n = 1148) of NCSP graduates passed the NMLE (*P*= 1.234). In Jiujiang, 93.7% (n = 1148) of CSP graduates and 89.0% (n = 189) NCSP graduates passed the NMLE (*P*= 1.181). In Gannan, CSP graduates (n = 1388, 90.6%) reported a significantly higher rate of passing the examination than NCSP graduates (n = 183, 86.5%, *P*= 1.002).

**Table 2:** Pass Rate of the China National Medical Licensing Examinations for Compulsory Services Program and Non-compulsory Services Program Graduates

**Schools**	**CSP**		**NCSP**		**Overall**	***P *** **Value**
	**N**	**%**	**N**	**%**		
Qinghai	268	76.79	101	76.52	76.72	.949
Guangxi	312	98.42	148	96.73	97.87	.234
Jiujiang	148	93.67	89	89.00	91.86	.181
Gannan	388	95.10	83	86.46	93.45	.002
Overall	1116	90.58	421	87.53	89.73	.153

Abbreviations: CSP, compulsory services program; NCSP, non-compulsory services program. Note: Due to COVID-19 pandemic, the China NMLE for 2020 has been delayed. Till the end of 2020 wave, graduates of 2019 have not participated in the examination yet, so only graduates of 2015-2018 are presented here.

###  Results of Job Performance Scale 

The total job performance score of CSP (Mean = 163.66) was basically the same with NCSP (Mean = 164.16) (see Table S3, Supplementary file 1). The differences in task performance, contextual performance, and learning performance were also nonsignificant between graduates of CSP and NCSP.

Table 3 displays that the job performance of both CSP and NCSP showed statistically significant differences among different medical universities, different years of graduation, sexuality, and marital status. For CSP graduates, the lowest job performance scale was in Qinghai (61.03) and highest in Guangxi (65.55, *P*< .001). CSP graduates in 2015 showed the highest score (66.47), while CSP graduates in 2019 presented the lowest score (62.07, *P*< .001). The mean score of male CSP graduates (64.70 was higher than that in their female counterparts (62.55, *P*< .001). Married CSP graduates had higher job performance score (65.36, *P*< .001) than those who were not married (Mean = 162.89, SD = 111.85, *P *<.001). CSP graduates working in public hospitals at county level and above, CHC or THC, and other places reported 62.26 (SD = 112.51), 63.79 (SD = 111.97), 64.44 (SD = 164.44) respectively in job performance scale (*P*= 1.372). About job-related characteristics, job performance of graduates of CSP was also significantly different concerning whether becoming attending physicians, whether working in hometown district, and whether obtaining formally funded positions. CSP graduates who have obtained formally funded positions obtained 63.76 in job performance scale (SD = 112.51, *P*= 1.038), higher than those who did not obtain (Mean = 159.78, SD = 111.57, *P*= 1.038). CSP graduates who have become attending physicians obtained 66.82 (SD = 112.76, *P*= 1.004) in job performance scale. The job performance score was 63.74 (SD = 111.99, *P*= 1.101) in CSP graduates who worked in contract signing places. CSP graduates who worked in hometown district presented higher job performance (Mean = 164.86, SD = 111.56, *P*< .001) than those who did not work in hometown district (Mean = 161.91, SD = 112.45, *P*< .001).

**Table 3:** Mean Score (SD) of the Job Performance Scale by Demographic and Job-Related Variables

**Characteristics**	**CSP (n = 1586)**	**NCSP (n = 568)**	**Overall (n = 2154)**	***P *** **Value**
School				
Qinghai	61.03 (13.27)	63.11 (10.85)	61.55 (12.58)	.033
Guangxi	65.55 (11.37)	62.36 (11.92)	64.60 (11.62)	.999
Jiujiang	65.54 (11.31)	67.90 (9.68)	66.47 (10.75)	.032
Gannan	64.11 (11.18)	64.62 (10.12)	64.20 (10.99)	.329
*P *value	<.001	<.001	<.001	
Year of graduation				
2015	66.47 (12.42)	66.13 (10.64)	66.33 (11.71)	.608
2016	65.62 (11.82)	65.85 (8.89)	65.68 (11.07)	.422
2017	63.15 (12.35)	61.79 (12.45)	62.82 (12.38)	.854
2018	62.08 (11.19)	64.21 (10.95)	62.55 (11.16)	.055
2019	62.07 (11.78)	61.27 (11.29)	61.92 (11.68)	.707
*P *value	<.001	<.001	<.001	
Demographic characteristics				
Female	62.55 (11.25)	62.92 (11.06)	62.65 (11.19)	.314
Male	64.70 (12.59)	65.50 (10.78)	64.90 (12.17)	.173
*P *value	<.001	0.005	<.001	
Not married	62.89 (11.85)	63.58 (11.38)	63.07 (11.73)	.156
Married	65.36 (12.25)	65.43 (10.01)	65.38 (11.66)	.474
*P *value	<.001	.063	<.001	
Current workplace				
Public hospitals at county level and above	62.26 (12.51)	64.20 (10.79)	63.80 (11.18)	.039
CHC and THC	63.79 (11.97)	62.58 (13.01)	63.78 (11.98)	.636
Other	64.44 (11.00)	64.22 (12.75)	64.25 (12.38)	.520
*P *value	.372	.881	.957	
Current job Information				
Obtaining formally funded positions	63.76 (12.51)	63.85 (11.04)	63.77 (11.90)	.462
Not obtaining formally funded positions	59.78 (11.57)	64.32 (10.98)	63.88 (11.11)	.007
*P *value	.038	.632	.864	
Becoming attending physicians	66.82 (12.76)	65.501 (9.19)	66.80 (12.68)	.558
Not becoming attending physicians	63.42 (11.92)	64.15 (11.00)	63.63 (11.68)	.101
*P *value	.004	.863	.005	
Working in contract signing place	63.74 (11.99)	-	63.74 (11.99)	-
Not working in contract signing place	60.37 (12.69)	-	60.37 (12.69)	-
*P *value	.101		.101	
Working in hometown district	64.86 (11.56)	-	64.86 (11.56)	-
Not working in hometown district	61.91 (12.45)	-	61.91 (12.45)	-
*P *value	<.001		<.001	

Abbreviations: SD, standard deviation; CSP, compulsory services program; NCSP, non-compulsory services program; CHC, community health center; THC, township health center. The total score of the job performance scale was 12 items × 7 points = 84.

CSP graduates did not had significantly lower job performance score than NCSP graduates in the results of *t *tests (*P *>.05), except that in Qinghai (61.03 vs. 63.11, *P*= 1.033) and Jiujiang (65.64 vs. 67.90, *P*= 1.032), those who worked in public hospitals at county level and above (62.26 vs. 64.20, *P*= 1.039), and those who did not obtain formally funded positions (59.78 vs. 64.32, *P*= 1.007).

After controlling for the covariates listed in Table 3, we found that there was no significant difference between two types of graduates in job performance in the multivariable regression analysis (Table 4). Combining CSP and NCSP graduates together as the total sample, the difference of job performance score between CSP and NCSP was -0.32 (standard error = 11.16), and did not show significant difference. This provides further evidence that although most medical graduates of CSP work in lower-level health facilities than NCSP, they do not show much difference in job performance. We further restricted the sample to graduates of 2015-2017 to rule out the effect of residency training. The difference of job performance score between CSP and NCSP was -1.36 (standard error = 11.53), and was not significant. We then conducted five robustness checks to examine the consistency of the results. We replaced the dependent variable with the log of the job performance score, divided the total job performance score into a dichotomous variable, and replaced the dependent variable with sub-dimensions of job performance of task, contextual, and learning performance (see Table S4-S6, Supplementary file 2). Overall, the results were quite robust. No significant difference was detected between CSP and NCSP in a log scale, logistic regression, and of sub-dimensions for job performance.

**Table 4:** Multivariable Regression on Total Job Performance in Compulsory Services Program and Non-compulsory Services Program Graduates

**Variables**	**Total Sample**		**Graduates of ** **2015-2017**		**CSP Sample**		**NCSP Sample**		**CSP Sample (Controlling for Contracting Strategy)**	
CSP	-0.32	(1.16)	-1.36	(1.53)						
Reference: Qinghai										
Guangxi	3.03***	(0.70)	2.16*	(0.95)	3.87***	(1.01)	-1.29	(1.21)	-3.15***^c^	(0.82)
Jiujiang	3.95***	(0.84)	3.15**	(1.05)	3.23**	(1.12)	4.39**	(1.33)		
Gannan	2.61***	(0.69)	2.38*	(0.94)	2.77**	(0.90)	0.91	(1.36)		
Reference: graduated in 2015										
Graduated in 2016	-0.23	(0.93)	-0.50	(0.95)	-0.62	(1.33)	0.28	(1.34)	-0.59	(1.33)
Graduated in 2017	-3.28***	(0.95)	-3.76***	(0.99)	-3.46**	(1.34)	-3.39*	(1.39)	-3.54**	(1.33)
Graduated in 2018	-2.72**	(1.00)			-3.53*	(1.38)	-1.46	(1.53)	-3.59**	(1.37)
Graduated in 2019	-3.10**	(1.01)			-3.26*	(1.38)	-4.07*	(1.63)	-3.27*	(1.37)
Male	1.94***	(0.50)	2.33***	(0.65)	1.85**	(0.60)	2.10*	(0.93)	1.79**	(0.60)
Married	1.25*	(0.59)	0.72	(0.67)	1.54*	(0.72)	0.51	(1.05)	1.47*	(0.71)
Formally funded positions	0.51	(0.92)	0.86	(1.15)	4.87*	(2.05)	-1.73	(0.99)	4.73*	(2.05)
Becoming attending physicians	-0.4	(1.36)	-0.46	(1.42)	-1.09	(1.63)	1.77	(7.74)	-0.93	(1.62)
Reference: working in public hospitals at county level and above										
Working in THC & CHC	0.19	(1.03)	1.41	(1.42)	-0.80	(1.17)	-3.60	(3.27)	-0.73	(1.17)
Working in other places	0.80	(1.63)	2.63	(2.18)	3.10	(4.05)	-0.45	(1.68)	3.20	(4.05)
Income of current job	0.01	(0.07)	-0.05	(0.08)	-0.04	(0.10)	0.13	(0.09)	-0.04	(0.10)
Working in contract signing place^a^					1.14	(2.12)			1.35	(2.11)
Working in hometown district^b^					1.29	(0.73)			1.48*	(0.70)
Constant	61.92***	(1.15)	62.55***	(1.33)	56.69***	(2.79)	63.68***	(1.51)	59.71***	(2.71)
R-sq	0.047		0.043		0.058		0.084		0.057	
N	2154		1322		1586		568		1586	

Abbreviations: CSP, compulsory services program; NCSP, non-compulsory services program; CHC, community health center; THC, township health center. Note: (1) Source: Compulsory Services Program 2020 Wave. (2) *** *P* <.001, ** *P* <.01, * *P* <.05; β coefficient was reported, with standard error in parenthesis. (3) Job income was measured in 1000 CNY. (4) ^ab^ These two variables are only for graduates of CSP. (5) ^c^ “Contracting strategy” is a dichotomous variable. It equals to 1 for Qinghai province where students choose contract signing places according to their rank of college entrance examination score, and 0 for other two provinces (Jiangxi province, which includes Jiujiang and Gannan; Guangxi province, which includes Guangxi) where students sign contracts with hometown counties in priority.

However, CSP and NCSP graduates did show different patterns of influencing factors. For graduates of CSP, whether obtaining formally funded positions was a significant indicator of their job performance. Graduates who obtained formally funded positions scored 4.87 (*P*< .05) higher on average than those who did not obtain. Longer working years are also associated with higher job performance. Compared with 2015 CSP graduates, 2016-2019 graduates all showed significantly lower job performance (β = 1-3.36, -3.53, -3.26, respectively). Male and married graduates also showed significantly higher job performance (β = 11.85, *P*< .01; β = 11.54, *P*< .05).

The contracting strategy implemented by Qinghai is different from that of the other two provinces. Qinghai allows students to choose contract signing places according to their rank of college entrance examination score within the province, while Guangxi and Jiangxi require students to sign contracts with hometown counties in priority. If their hometowns cannot accept more CSP students or the candidates exceed the primary health institutions’ recruiting capacity, students will be transferred to neighboring counties. Since Qinghai adopted different contracting strategy while the strategy is similar in the other two provinces, we separately controlled the different contract signing strategy in the regression for CSP (Table 4). We found that “working in hometown district” became significant, indicating that working in hometown might increase job performance (β = 11.48, *P*< .05).

## Discussion

This study evaluated the job performance of medical graduates trained by CSP, while most existing studies focused on attraction and retention.^20,51-54^ To the best of our knowledge, this study is the first study to examine the job performance of CSP graduates in China. We found that CSP graduates have demonstrated good job performance in primary health settings in China. Although CSP students were recruited with lower college entrance examination scores, the pass rate of NLME and self-reported job performance scale both suggested that CSP graduates exhibited as good job performance as NCSP peers who performed better in college entrance examination. Our results were consistent with previous studies. Putthasri et al also found that in Thailand, graduates from special track (similar to CSP) had higher clinical competencies than those from normal track (similar to NCSP).^24^

International CSP experiences confirmed that students improved academic performance during school. In Japan, Matsumoto and colleagues found that students increased their academic standing throughout undergraduate education.^55^ In the United States, Rabinowitz et al also found that although students had lower test scores than non-program peers of the same medical schools when matriculated, the difference decreased during medical education and disappeared by graduation.^56^ We further confirmed that such positive changes persisted after students graduated and continued after they began practicing medicine. Our study suggested that CSP graduates were well-trained and had the competency to become qualified workforces in primary health systems.

Currently, most rural primary health workers in China have only received limited medical training and lower qualification. More than 1/3 physicians in THC did not take the NMLE and most are three-year and less medical graduates.^57^ Without CSP, those well-trained five-year medical graduates would not work in rural or remote areas due to lower income, limited career opportunities and other limitations. Since the college entrance scores of CSP students on matriculation were lower than that of NCSP students. People would concern whether their job performance and the quality of health services they provide is also lower than that of NCSP. However, our results suggested that there was no significant difference between the two types of graduates, indicating that after five-year undergraduate education, the quality and competency of CSP graduates were assured; this likely came from standardized residency training received. China introduced the residency training policy for CSP graduates in 2015, aiming to improve the medical education quality of CSP.^58^ Through comprehensive hospital, THC and CHC internship during undergraduate study and residency training after graduation, CSP graduates were exposed to real working conditions and improve clinical capability and service skills; this meant that they could increase the accessibility of equal-quality or high-quality services in rural areas, and could also help to address the deficit and maldistribution of health workforce and improve health equity in rural and remote areas.

As for the influencing factors, formally funded positions and working in hometown had positive influence on job performance. Formally funded positions could increase employees’ recognition towards the organization and maintain close contact between employees and employers.^59^ The national policies promised to provide formally funded positions for CSP graduates, which was also the primary attraction for students’ recruitment; but for most NCSP graduates who were based in tertiary hospitals in cities, occupational mobility was important for their future career development. Therefore, other factors like levels of hospital and income could be more important than stable formally funded positions which restricted mobility. However, due to limitations in power decentralization, THC cannot fully decide their formally funded positions when recruiting new employees.^35^ The positions may hang for a long time, discouraging CSP graduates’ working enthusiasm, especially for newly graduated students who showed lower job performance compared to those who have worked for a longer period. The first few years of working in rural areas could be critical for career development and future retention. Before formally funded positions fulfill, the health organizations and local governments should provide more support to enhance job performance to secure service quality, as well as increase retention in the long run. The local health governments should clarify the responsibilities of contract period, notify the contracted organizations timely and supervise the reception process to guarantee they settle down quickly. Especially, since formally funded positions were important for job performance of CSP graduates, the provincial and municipal policy-makers should press to accelerate the process of providing the positions.^36^ The contracted organizations should provide occupational and life guidance to CSP graduates to improve their understanding of the work in PHC and help them adapt to local living environment and culture; for example, an experienced doctor could be arranged as a mentor for each CSP graduate in the first year.

Working in hometown could also increase the job performance of CSP graduates. “Whether working in hometown” was insignificant in multivariable regression, but became significant again when we controlled for the contracting strategies. In Qinghai, the contract signing places were not related to students’ hometown necessarily, because the top-ranked students will give priority to developed areas with lower altitude, even if they came from higher-altitude and less developed areas. Signing contracts with hometowns was not always a desired option. As a result, low-ranked students may be transferred to less desirable working places after graduation; their job performance may decrease. While in Jiangxi and Guangxi, as stipulated by the policies, students’ primary contract signing places were their hometowns. Allowing CSP graduates to choose where they sign contracts with by rank of score could have negative influence on job performance, especially for those with lower-rank who had to be allocated to poorer areas. Some studies have shown that hometown recruiting can increase the job satisfaction and probability of retention in rural health institutions.^16^ However, there is limited evidence about which way of signing contracts will predict better job performance outcome. Our results suggested that signing contracts with hometown might have positive impact on students’ job performance; maintain high job performance would bring higher satisfaction of their job, which might increase their retention in the long run. We suggested that CSP graduates should sign contracts with hometowns or places close to hometowns first, even for less developed places like Qinghai; besides, allocating students by rank was unequal to less developed places and against the goal of CSP.

 This study has several limitations. First, this study used self-reported data to measure the job performance. It might be subject to self-report bias to distinguish the two types of medical graduates. With more resources and time, interviewing the graduates in their workplace, their workmates and patients would bring us more findings about their job performance. Examining their medical records and compared with NCSP graduates would also help us to quantify their job performance. Second, the attrition of NCSP graduates was higher than that of CSP. NCSP graduates had more diversified employment, making them difficult to follow up. The different attrition rates might bring bias to the results. Third, due to the pandemic of coronavirus disease 2019 (COVID-19), the NMLE in 2020 has been delayed. Most students graduated in 2019 had not taken the exam by the time when we conducted the 2020 follow-up survey. We can only obtain the pass rate of the NMLE for graduates in 2015, 2016, 2017, and 2018, which might affect the results.

## Conclusion

 Overall, CSP graduates have demonstrated as good job performance as their NCSP peers; they did not have lower pass rate of NMLE, and did not have lower job performance score even if they lagged behind when enrolment. CSP graduates showed the potential competence to provide high-quality care to a large population in rural areas in China. Whether working in hometowns and obtaining formally funded positions can have positive influence on job performance. Contracted organizations should provide early career support like occupational and life guidance for CSP graduates, especially in the first few years after graduation. Policy-makers should accelerate providing formally funded positions and prioritize signing contracts with hometowns or places close to hometowns.

## Acknowledgements

 The authors want to thank Qinghai University, Jiujiang University, Gannan Medical University, and Guangxi Medical University for their support in the establishment of the cohorts.

## Ethical issues

 The study has been approved by the Institutional Review Board of Peking University (IRB00001052-15027). Informed consent was obtained from all participants prior to questionnaire administration.

## Competing interests

 Authors declare that they have no competing interests.

## Authors’ contributions

 ML drafted the manuscript, and conducted the data analysis and interpretation. BZ, TW, DH collected and managed the data. XL and ZW designed the study, managed the data, and made critical interpretations and revisions on some intellectual contents of the article. All authors gave final approval for the final version to be published.

## Supplementary files


Supplementary file 1. Response Rate of the Survey and Job Performance Scale.

Supplementary file 2. Robustness Check for Multivariable Regressions on Total Job Performance.
