# Supplementary material for: Job Performance of Medical Graduates With Compulsory Services in Underserved Rural Areas in China: A Cohort Study
Source: Int J Health Policy Manag. 2022 Feb 14;11(11):2600–9. doi: 10.34172/ijhpm.2022.6335 (PMC9818097; doi:10.34172/ijhpm.2022.6335)
Supplement: Supplementary file 1 — Response Rate of the Survey and Job Performance Scale. [file ijhpm-11-2600-s001.pdf]

Article title: Job Performance of Medical Graduates With Compulsory Services in Underserved Rural Areas in China: A Cohort Study

Journal name: International Journal of Health Policy and Management (IJHPM)

Authors' information: Mingyue Li[1,2] , Ziyue Wang[2], Baisong Zhang[1,2], Tiantian Wei[1,2], Dan Hu[2], Xiaoyun Liu[2]*

[1]Department of Health Policy and Management, School of Public Health, Peking University, Beijing, China.

[2]China Center for Health Development Studies, Peking University, Beijing, China.

(*Corresponding author: xiaoyunliu@pku.edu.cn)

Supplementary file 1. Response Rate of the Survey and Job Performance Scale

Table S1. Dimensions, definitions, and reliability of the job performance scale

Dimensions	Definition	Cronbach's α	Cronbach's α in literature
Task performance	Related to work output and directly serving organizational goals，reflected in work quality, efficiency, ability and input	0.947	0.805
Learning performance	The process that individuals form learning plans and apply new knowledge and skills to the changing organizational environment, including learning desires, behavior and result application	0.954	0.842
Contextual performance	Indirect assistance to the realization of organizational goals, efforts to improve the harmony of working environment, including assisting colleagues, abiding by rules, extra efforts, personal self-discipline, supporting organizational goals, etc.	0.936	0.714
Total	-	0.897	-

Table S2. The response rate for CSP and NCSP graduates in 2020 wave

Years of graduation	CSP			NCSP		
	Baseline N	2020 wave N	Response rate %	Baseline N	2020 wave N	Response rate %
2015	305	239	78.4%	315	181	57.5%
2016	437	330	75.5%	302	163	54.0%
2017	481	383	79.6%	330	203	61.5%
2018	437	328	75.1%	312	201	64.4%
2019	381	351	92.1%	320	261	81.6%

Abbreviations: CSP, compulsory services program; NCSP, non-compulsory services program;
The 2020 wave is the fourth, the third, the second and the first follow-up for 2015, 2016, 2017, 2018, and 2019 graduates, respectively.

Table S3. Mean score (SD) of each item in the 7-point Likert job performance scale

Dimensions	Items[a]	CSP	NCSP
Task performance	Total	21.12 (4.35)	21.50 (3.96)
	I can always finish my work in time skillfully	5.38 (1.20)	5.46 (1.12)
	I can always finish my work with high quality according to the requirements of performance appraisal	5.31 (1.23)	5.42 (1.08)
	I have very good job-related knowledge and skills	5.23 (1.22)	5.25 (1.08)
	I always put all my energy into my work	5.20 (1.26)	5.37 (1.15)
Contextual performance	Total	26.45 (5.32)	26.61 (4.81)
	I often offer help to other colleagues	5.39 (1.22)	5.51 (1.12)
	I can always strictly abide by the rules and regulations of the institution	6.04 (1.13)	6.04 (1.05)
	Even without the supervision of the leader, I can always work with enthusiasm	5.51 (1.26)	5.68 (1.12)
	I will volunteer to take on some responsibilities that are not my own	4.88 (1.43)	4.93 (1.36)
	I will provide some good suggestions of the management decisions for employers	4.63 (1.55)	4.45 (1.52)
Learning performance	Total	16.10 (3.47)	16.06 (3.28)
	I attach great importance to learning and have a strong desire to learn	5.54 (1.24)	5.37 (1.21)
	I have learned a lot of new working knowledge and skills through various channels	5.30 (1.28)	5.32 (1.15)
	I have greatly improved my personal ability through study and used it in my work	5.25 (1.28)	5.36 (1.12)
	Total	63.66 (12.01)	64.16 (10.99)

Abbreviations: CSP, compulsory services program; NCSP, non-compulsory services program.
[a]For each item, the options are as follows: 1=never, 2=a few times a year or less, 3=once a month, 4=a few times a year, and 5=once a week, 6=a few times a week; 7=every day. The corresponding score ranges from 1 to 7.
