# Supplementary material for: Job Performance of Medical Graduates With Compulsory Services in Underserved Rural Areas in China: A Cohort Study
Source: Int J Health Policy Manag. 2022 Feb 14;11(11):2600–9. doi: 10.34172/ijhpm.2022.6335 (PMC9818097; doi:10.34172/ijhpm.2022.6335)
Supplement: Supplementary file 2 — Robustness Check for Multivariable Regressions on Total Job Performance. [file ijhpm-11-2600-s002.pdf]

Article title: Job Performance of Medical Graduates With Compulsory Services in Underserved Rural Areas in China: A Cohort Study

Journal name: International Journal of Health Policy and Management (IJHPM)

Authors' information: Mingyue Li[1,2] , Ziyue Wang[2], Baisong Zhang[1,2], Tiantian Wei[1,2], Dan Hu[2], Xiaoyun Liu[2]*

[1]Department of Health Policy and Management, School of Public Health, Peking University, Beijing, China.

[2]China Center for Health Development Studies, Peking University, Beijing, China.

(*Corresponding author: xiaoyunliu@pku.edu.cn)

Supplementary file 2. Robustness Check for Multivariable Regressions on Total

Job Performance

Table S4. Multivariable regression on total job performance in CSP and NCSP graduates

Variables	β coefficient (Standard error)	
	Log total performance	
CSP	-0.01	(0.02)
Reference: Qinghai		
Guangxi	0.05***	(0.01)
Jiujiang	0.07***	(0.02)
Gannan	0.05***	(0.01)
Reference: graduated in 2015		
Graduated in 2016	0.00	(0.02)
Graduated in 2017	-0.06**	(0.02)
Graduated in 2018	-0.04*	(0.02)
Graduated in 2019	-0.05*	(0.02)
Male	0.03**	(0.01)
Married	0.02*	(0.01)
Officially budgeted posts	0.01	(0.02)
Becoming attending physicians	-0.01	(0.03)
Reference: working in public hospitals at county level and above		

Working in THC & CHC	0.00	(0.02)
Working in other places	0.01	(0.03)
Income of current job	0.00	0.00
Working in contract signing place[a]		
Working in hometown district[b]		
Constant	4.10***	(0.02)
R-sq	0.040	
N	2154	

Note: (1) Source: Compulsory Services Program 2020 Wave. (2) *** p<0.001, ** p<0.01, * p<0.05; standard error in parenthesis. (3) Job income was measured in 1,000 CNY. (4) [ab]These two variables are only for graduates of CSP.

Table S5. Logistic regressions on total job performance

Variables	Odds Ratio (95% Confidence Interval)		
	Total performance	Total performance in CSP	Total performance in NCSP
CSP	0.895	-	-
	(0.569 - 1.408)	-	-
**Reference: Qinghai**			
Guangxi	1.323**	1.483**	0.808
	(1.002 - 1.747)	(1.011 - 2.176)	(0.471 - 1.387)
Jiujiang	1.627***	1.394	1.976**
	(1.181 - 2.240)	(0.917 - 2.120)	(1.154 - 3.383)
Gannan	0.991	1.027	0.840
	(0.753 - 1.303)	(0.727 - 1.452)	(0.456 - 1.549)
**Reference: graduated in 2015**			
Graduated in 2016	0.725*	0.841	0.578*
	(0.518 - 1.013)	(0.523 - 1.351)	(0.333 - 1.001)
Graduated in 2017	0.494***	0.537**	0.432***
	(0.347 - 0.703)	(0.330 - 0.873)	(0.241 - 0.772)
Graduated in 2018	0.493***	0.498***	0.566*

	(0.335 - 0.724)	(0.297 - 0.835)	(0.301 - 1.065)
Graduated in 2019	0.419***	0.491***	0.274***
	(0.283 - 0.619)	(0.293 - 0.824)	(0.131 - 0.573)
Male	1.739***	1.895***	1.454*
	(1.427 - 2.120)	(1.502 - 2.391)	(0.972 - 2.176)
Married	1.129	1.269*	0.843
	(0.901 - 1.415)	(0.969 - 1.662)	(0.548 - 1.298)
Formally funded positions	0.985	4.148**	0.650*
	(0.697 - 1.390)	(1.401 - 12.281)	(0.422 - 1.002)
Becoming Attending physicians	0.923	0.949	1.921
	(0.567 - 1.502)	(0.535 - 1.682)	(0.096 - 38.539)
**Reference: Working in public hospitals at county level and above**			
Working in THC & CHC	1.395	1.104	0.893
	(0.910 - 2.139)	(0.681 - 1.789)	(0.268 - 2.973)
Working in other places	1.295	1.107	1.193
	(0.683 - 2.458)	(0.212 - 5.792)	(0.565 - 2.522)
Income of current job	0.994	0.985	1.011
	(0.969 - 1.021)	(0.957 - 1.013)	(0.975 - 1.048)
Working in contract signing place[a]	-	0.706	-
		(0.315 - 1.583)	
Working in hometown district[b]	-	1.164	-
		(0.876 - 1.547)	
Constant	0.341***	0.100***	0.546*
	(0.216 - 0.537)	(0.025 - 0.400)	(0.298 - 1.000)
Observations	2,154	1,586	568

Notes: (1) In this logistic regression, we divided job performance score into a dichotomous variable (those obtaining 6 or 7 for each item with total score being or above 72 were identified as high work performance). (2) Source: Compulsory Services Program 2020 Wave (3) *** p<0.01, ** p<0.05, * p<0.1, robust CI in parentheses; (4) Job income was measured in 1,000 CNY. (5) [ab]These two variables are only for graduates of CSP.

Table S6. Multivariable regressions on three sub-dimensions of job performance

Variables	β coefficient (Standard error)					
	Learning performance		Contextual performance		Task performance	
CSP	0.25	(0.34)	-0.25	(0.52)	-0.31	(0.41)
**Reference: Qinghai**						
Guangxi	0.77***	(0.21)	1.16***	(0.31)	1.10***	(0.25)
Jiujiang	0.98***	(0.25)	1.33***	(0.38)	1.64***	(0.30)
Gannan	0.71***	(0.20)	0.70*	(0.31)	1.20***	(0.25)
**Reference: graduated in 2015**						
Graduated in 2016	0.21	(0.27)	-0.29	(0.41)	-0.15	(0.33)
Graduated in 2017	-0.54	(0.28)	-1.16**	(0.42)	-1.58***	(0.34)
Graduated in 2018	-0.37	(0.29)	-1.02*	(0.45)	-1.34***	(0.36)
Graduated in 2019	-0.62*	(0.30)	-1.10*	(0.45)	-1.38***	(0.36)
Male	0.53***	(0.15)	0.57*	(0.22)	0.84***	(0.18)
Married	0.12	(0.17)	0.61*	(0.26)	0.52*	(0.21)
Formally funded positions	-0.14	(0.27)	0.38	(0.41)	0.26	(0.33)
Becoming Attending physicians	-0.10	(0.40)	-0.27	(0.61)	-0.03	(0.49)
**Reference: Working in public hospitals at county level and above**						
Working in THC & CHC	0.01	(0.30)	0.16	(0.46)	0.02	(0.37)
Working in other places	0.16	(0.48)	0.25	(0.73)	0.39	(0.58)
Income of current job	0.00	(0.02)	0.01	(0.03)	-0.01	(0.02)
Constant	15.42***	(0.34)	25.78***	(0.51)	20.72***	(0.41)
R-sq	0.031		0.029		0.071	
Observations	2154		2154		2154	

(1) Source: Compulsory Services Program 2020 Wave (2) *** p<0.01, ** p<0.05, * p<0.1(4) Job income was measured in 1,000 CNY. (5) [ab]These two variables are only for graduates of CSP.
